# Adult-onset hypopigmented mycosis fungoides: CD8-predominant presentation beyond the classic patch

**DOI:** 10.1016/j.jdcr.2026.04.008

**Published:** 2026-04-17

**Authors:** Aashna Farishta, Olga Gomeniouk, Rohan Mital, Vida Ehyaee, Smita Aggarwal, Mitul B. Modi

**Affiliations:** a Rush Medical College, Chicago, Illinois; b Department of Dermatology, Rush University Medical Center, Chicago, Illinois; c Department of Pathology, University of Michigan, Ann Arbor, Michigan; d Division of Dermatopathology, Department of Dermatology, Rush University Medical Center, Chicago, Illinois

**Keywords:** cutaneous t-cell lymphoma, epidermotropism, hypopigmented mycosis fungoides, hypopigmented patches, narrow-band UVB phototherapy

## Introduction

Mycosis fungoides (MF) is the most common type of cutaneous T-cell lymphoma, and hypopigmented mycosis fungoides (HMF) is a rare clinical variant most often seen in children, adolescents, and young adults. It commonly presents with hypopigmented patches or thin plaques with a fine scale, mimicking benign dermatoses. These nonspecific features frequently lead to a delayed diagnosis.

Histologically, HMF demonstrates epidermotropism with haloed, atypical lymphocytes and sparse dermal infiltrate, often accompanied by melanin incontinence, slight psoriasiform hyperplasia, and occasional interface-like changes or folliculotropism.^1^^,^^2^ These features overlap with classical MF but display more prominent epidermotropism and pigmentary alterations. Immunophenotypically, it often shows a predominance of CD8+ cytotoxic T cells, in contrast to the CD4+ predominance typical of classic MF.^3^

The pathogenesis of hypopigmentation observed in HMF results from direct cytotoxic CD8+ malignant T cells infiltrating the epidermis and directly damaging melanocytes, reducing their number and impairing melanin synthesis through loss of key melanocytic markers and cytokine-mediated dysfunction.^1^^,^^4^^,^^5^ The recognition of HMF across a broader age spectrum is key to avoid a missed or delayed diagnosis in older patients, in whom pigmented variants may be overlooked.

## Case presentation

**Fig 1:**
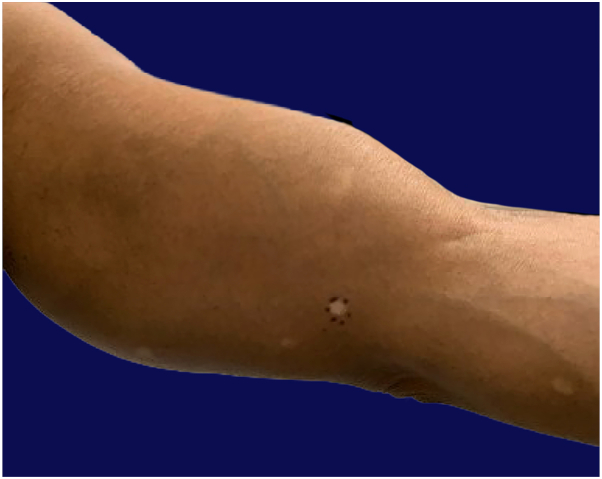
Hypopigmented mycosis fungoides. Multiple hypopigmented to depigmented macules and patches involving the bilateral inner arms.

A 50-year-old patient presented with a 6 to 9-m history of progressive, asymptomatic, hypopigmented patches localized to the bilateral inner arms (Fig 1). The patient denied pruritus, pain, or preceding inflammation. No personal or family history of autoimmune disease, cutaneous lymphoma or photosensitivity was reported.

**Fig 2:**
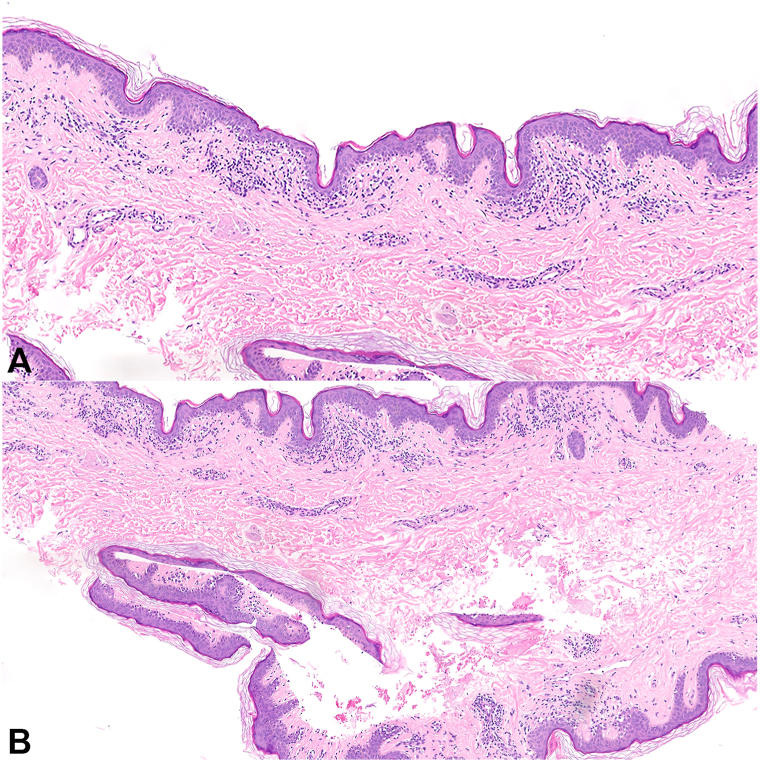
Hypopigmented mycosis fungoides H&E sections from a shave biopsy of the left inner arm showing epidermal atrophy, a lichenoid lymphocytic infiltrate, and pigment incontinence (**A,** 200×; **B,** 250×).

A shave biopsy from the left inner arm demonstrated epidermal atrophy, lichenoid lymphocytic infiltrate at the dermoepidermal junction, papillary dermal edema, upper dermal sclerosis, and marked pigment incontinence. Atypical junctional lymphocytes were moderate-to-large in size with perinuclear halos (Fig 2).

**Fig 3:**
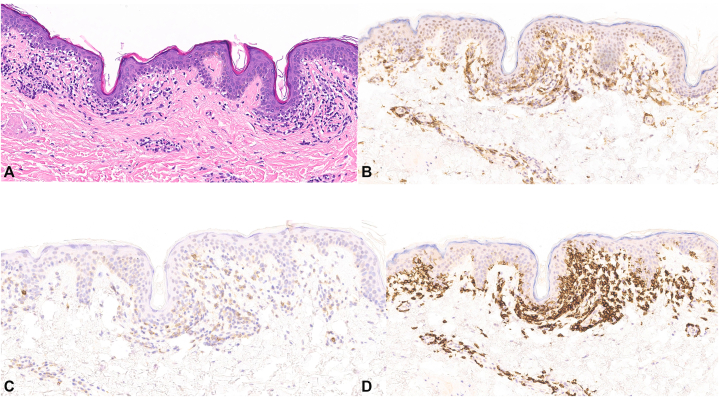
Hypopigmented mycosis fungoides Immunohistochemistry demonstrates a CD8-predominant epidermotropic T-cell infiltrate (**A,** H&E, 100×; **B,** CD4, 100×; **C,** CD7, 100×; **D,** CD8, 100×).

Immunohistochemistry revealed a dense dermal and epidermal T-cell infiltrate positive for CD3^+^, with a reversed CD4:CD8 ratio with predominance of CD8^+^ cells. CD5 expression was preserved, while CD7 expression was markedly reduced. Rare B lymphocytes were identified with CD20 staining. SOX10 confirmed preserved melanocytes, and Fontana-Masson stain demonstrated basal melanin deposition (Fig 3).

T-cell receptor gene rearrangement studies were negative for clonality. Given the clinical, histopathologic, and immunophenotypic findings, a diagnosis of CD8-predominant hypopigmented MF was established. The patient was treated with narrow-band ultraviolet B (NB-UVB) phototherapy, with complete clinical clearance of the hypopigmented patches.

## Discussion

This case highlights an uncommon CD8+-predominant hypopigmented MF in a middle-aged patient, broadening the demographic profile traditionally associated with this clinical variant of MF. Most reports describe cases in the pediatric and young adult population, underscoring the need for clinicians to maintain a high index of suspicion for HMF even in populations where it is considered atypical.

The patient’s lesions were localized, nonpruritic, and lacked preceding inflammation, features that are in line with benign hypopigmentary disorders. However, histopathology revealed lichenoid lymphocytic infiltrate and pigment incontinence, findings consistent with HMF. Additionally, SOX10 confirmed preserved melanocytes, while Fontana-Masson staining demonstrated basal melanin. The immunoprofile revealed a reversed CD4:CD8 ratio, a characteristic feature of HMF. CD4 predominance is typically seen in classic MF, while CD8 predominance is characteristic for HMF and may contribute to the hypopigmented phenotype by direct cytotoxic effects on melanocytes. Although TCR gene rearrangement studies were negative for clonality in this case, the absence of detectable clonality does not exclude MF in its early patch stage.^6^ Thus, diagnosis must rely on the integration of clinical, histopathologic, and immunophenotypic findings.

The prognosis of HMF is typically favorable, including in older adults, as most cases present at early stages with an indolent course.^2^^,^^7^ Pediatric and young adult cases are more frequently reported, with long-term studies confirming their indolent nature.^2^ Fewer adult-onset cases have been described, but available reports suggest a similarly favorable prognosis.^7^ Notably, the CD8+ cytotoxic phenotype is generally linked to less aggressive clinical behavior compared to classic MF.^8^ Early recognition is critical, as delayed diagnosis may result in patients receiving inappropriate treatments for benign dermatoses.

From a therapeutic standpoint, given the early patch-stage presentation and favorable prognosis of hypopigmented mycosis fungoides, skin-directed therapies remain the standard of care. NB-UVB phototherapy is generally preferred due to its safety, convenience and efficacy in inducing repigmentation.^9^^,^^10^ Psoralen plus UVA (PUVA) may be considered for thicker plaques or refractory disease, though it carries a greater risk of phototoxicity and long-term carcinogenesis.^10^ Long-term follow-up is essential, as relapses are common despite the overall favorable outcome.

## Conflicts of interest

None disclosed.
